# Relationship between Serum Osteocalcin and Carotid Atherosclerosis in Middle-Aged Men in China: A Cross-Sectional Study

**DOI:** 10.1155/2018/1751905

**Published:** 2018-08-14

**Authors:** Hui Deng, Hao Lu, Yang Dai, Lingling Li, Juan Cao, Dalong Zhu

**Affiliations:** ^1^Department of Geriatrics, Drum Tower Clinical Medical College of Nanjing Medical University, China; ^2^Department of Endocrinology, Drum Tower Clinical Medical College of Nanjing Medical University, China

## Abstract

**Background and Purpose:**

Numerous investigations found that there exists a close relationship between serum osteocalcin and incurrence of atherosclerosis, but studies investigating the effect of serum osteocalcin on carotid atherosclerosis are very limited. Our study is aimed at investigating the role of osteocalcin in carotid atherosclerosis in middle-aged men in China.

**Methods:**

A total of 335 male middle-aged participants (40-60, 52.4 ± 3.87 years averagely) were enrolled from the institute. The carotid intima-media thicknesses (CIMT) of each subject were measured. All subjects were included either in the carotid atherosclerosis (AS) group or the control group according to the CAS diagnosis criteria. Serum osteocalcin and other markers of each patient were assessed following standard laboratory tests protocol.

**Results:**

135 subjects were included in AS group, and 199 subjects were included in control group. The average osteocalcin level in AS group was 18.71 ± 6.20 ng/ml and was significantly different from that in control group, which was 20.38 ± 7.19 ng/ml (p=0.039). Logistic regression analysis revealed that OCN (standardized *β*=-0.024, p=0.013) and FINS (standardized *β*=-0.065, p=0.010) were independently and inversely associated with the carotid atherosclerosis, while FPG (standardized *β*=0.538, p=0.006) was independently and positively associated with the carotid atherosclerosis.

**Conclusion:**

Our study suggests that OCN is independently related to carotid atherosclerosis in middle-aged male individuals.

## 1. Introduction

Atherosclerosis-induced cardiovascular events and cerebrovascular events cause death in almost 50% of cases in developed countries [1]. Of these fatal cases, 20% are related to carotid artery [2]. Patients with concomitant carotid atherosclerotic disease and coronary artery disease are more likely to have widespread atherosclerosis and are related to higher risk of recurrent symptoms and complications [3]. Several risk factors of carotid atherosclerotic disease, including smoking, age, hypertension, and abnormal levels of triglyceride and lipoprotein cholesterol, have been identified [4]. Notably, aggregating evidence demonstrated that the diabetes mellitus is an predicator of atherosclerosis [5].

Recently an increasing number of investigations found that bone metabolism could be related to carotid atherosclerosis. Carotid intima-media thickness (CIMT), known as a significant factor to increase the risk of myocardial infarction and ischemic stroke [6], was also reported to be closely related to lumbar spine BMD in postmenopausal women [7]. Atherogenic stimuli not only induce the transformation of smooth cells into osteoblast-like cells and acceleration of vascular calcification, but also prompt the release of bone formation markers and osteoclast differentiation and activation [8].

Osteocalcin (OCN), for example, a bone formation marker which is produced by osteoblastic cells and derived from procollagen metabolism, is a specific and sensitive parameter of bone remodeling involved in bone mineralization and calcium homeostasis [9]. OCN is reported to participate in the regulation of the adipose-related gene expression and influence the glucose tolerance, fat consumption, and insulin resistance [10]. Previous studies also found that bone remodeling could be regulated by adipose tissues through the effects of leptin on osteoblasts [11], and therefore a feedback loop with bone-regulated energy metabolism, and that bone in skeleton is indicated to be an endocrine organ.

Previously, the level of serum OCN was found to be closely related to the participants who had self-reported cardiovascular disease in middle and elderly aged Chinese [12]. Similarly, OCN is also found to be a critical determinant of coronary atherosclerotic severity in Chinese male participants [13]. On this basis, it is reasonable to deduce that serum OCN is a potential candidate for detecting the risk of carotid atherosclerosis and the abnormal glucose metabolism. In our study, to address this issue, we collected anthropometric parameters, serum OCN level, blood glucose, and lipoid profile in a total of 355 middle age (40-60 years) men who were divided into AS group and control group according to the measurement of carotid intima-media thicknesses (CIMT), and their association with carotid atherosclerosis was analyzed.

## 2. Materials and Methods

### 2.1. Study Design and Study Objects

Between January 2014 and December 2016, male patients aged between 40 and 60 years who underwent routine medical health examination in Nanjing Drum Tower Hospital were screened and selected for our study. Our study was approved by the ethics committee of the institute and the informed consent was taken from all the patients. The physical characteristics of each patient including age, BMI, and waistline were recorded. Their history of alcohol consumption, current cigarette smoking, hypertensive disease, diabetes mellitus, bone fracture, and operations was recorded. Active smokers were defined as smoking more than 10 cigarettes per day. Systolic blood pressure of 140 mmHg and/or diastolic blood pressure of 90 mmHg was defined as arterial hypertension [14].

Patients included in our study were either assigned to atherosclerotic (AS) group or control group according to the CAS diagnosis criteria; e.g., the intima-media thickness is larger than 1.0 mm regardless of the presence of the atherosclerotic plaques. Female patients were not included in order to avoid the influences on bone turnover markers caused by the changes of hormone level during perimenopausal period. Patients who had renal dysfunction, rheumatoid diseases, thyroid dysfunction, parathyroid dysfunction, adrenal dysfunction, and administration of bisphosphonates, vitamin D, calcium and derivatives, or hormonal and diuretic drugs were excluded in our study. Moreover, patients with tumor, long-term bedridden treatment, previous fragility fracture [15] within a maximum of 1 year, and addiction to alcohol, smoking, or coffee were also excluded in our study. Eventually, a total of 335 consecutive patients are included in our study, including 136 patients in AS group and 199 patients in control group.

### 2.2. Biochemical Analysis

After overnight fasting for a minimum of 12 hours, the blood samples were collected from each subject in the morning local time and transferred to serum separation tubes. After centrifugation, serums were obtained and were used for determining serum level of OCN. Specifically, hundreds of tests were uniformly conducted in the laboratory of the institute utilizing a computer automatically controlled analyzer for quantitively testing the OCN with reagent kit, and the whole procedures were strictly conducted by the same group of technicians according to the manufacturer's manuals. On the other hand, liver and renal function markers, serum calcium, phosphorus, and alkaline phosphatase levels were also detected. The above test results regarding AS group and control group were further compared and analyzed.

### 2.3. Statistical Analysis

All data were expressed as means ± standard deviation (SD) or standard error (SE). All statistical analyses were performed using the SPSS 19.0 program, and significance level of p < 0.05 was used for all comparisons. Comparisons of OCN level and other serum metabolic markers compared between AS group and control group were assessed by the nonparametric Mann–Whitney U test. The correlation between significantly different markers and AS risk was analyzed by Spearman tests and logistic regression.

## 3. Results

### 3.1. The Characteristics of Study Population

OCN was nonnormally distributed. The median of OCN was 17.87 (95% CI: 17.66 to 19.76) in AS group and 18.98 (95% CI: 19.38 to 21.39) in control group and was found to be significantly less in AS group than that in control group (18.71 ± 6.20 versus 20.38 ±7.19, p= 0.039). The mean data and standard deviation could be seen in Table 3.

The demographic and clinical characteristics of each subject in AS group and control group are presented in Table 1. The age, SBP and DBP, and BMI were compared, and no significant differences were found between two groups. There was also no significant difference found in the prevalence of smokers, hypertension, and smoking cases between AS group and control group (as shown in Table 2). Similarly, there was no significant difference between AS groups and control groups, regarding renal function markers (e.g., BUN, creatinine), some lipid markers (e.g., triglyceride, total cholesterol, and LDL-C), and other makers such as calcium and phosphorus. However, the FPG in AS group was found to be significantly higher than that in control group (5.81 ± 1.52 versus 5.32 ± 0.98, p=0.004), and the HbAlc was also found to be significantly higher in the AS group than control group (5.88 ± 0.88 versus 5.63 ± 0.67, p=0.008), indicating an impaired glucose tolerance. Moreover, the HDL-C was found to be significantly higher in control group than in AS group, implying a better inhibition to oxidation, coagulation, and platelet aggregation in nonatherosclerotic individuals [16].

### 3.2. Association of OCN with Other Metabiotic Markers in AS Group and Control Group

**Figure 1:**
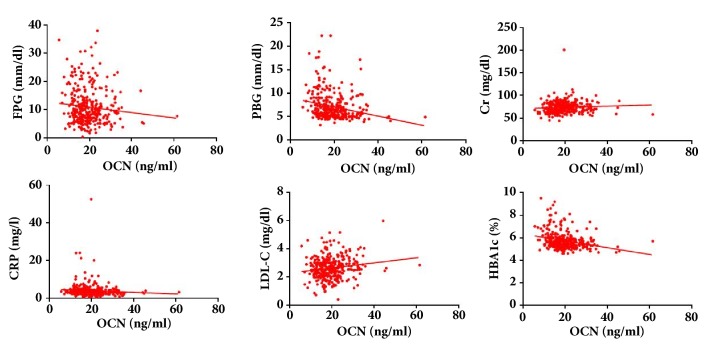
The correlation between OCN and FPG, PBG, Cr., CRP, LDL-C, and HbA1c.

The association between OCN and glucose metabolic markers were analyzed. In AS group, Spearman's correlation analysis demonstrated a negative correlation between OCN and FPG (r = -0.389, p <0.001), OCN and HbA1c (r = -0.306, p <0.001), and OCN and PBG (r = -0.365, p <0.001). In control group, Spearman's correlation analysis demonstrated a negative correlation between OCN and FPG (r = -0.192, p = 0.007), OCN and HbA1c (r = -0.199, p = 0.005), OCN and PBG (r = -0.147, p = 0.039), and OCN and FINS (r = -0.143, p = 0.043). Moreover, the association between OCN and other risk factors such as age, BMI, SBP, DBP, HbA1c, creatinine, and total cholesterol was also investigated. In AS group, Spearman's correlation analysis demonstrated a significant correlation between OCN and age (r = 0.186, p = 0.030) and OCN and SBP (r = -0.156, p = 0.070). However, in control group, Spearman's correlation analysis demonstrated no significant correlation between OCN and other risk factors. On the other hand, as shown in Table 3, in whole study population, OCN was found to have significant positive correlation with creatine and LDL-C, and negative correlation with FPG, PBG, CRP, and HbA1c (Figure 1).

A logistic regression analysis was conducted to identify the independent association between levels of OCN, traditional atherosclerotic risk factors as well as glucose metabolic markers (including age, BMI, DM, hypertension, SBP, DBP, HbA1c, total cholesterol, creatinine, FPG, PBG, and FINS), and carotid atherosclerosis, and odds ratios (OR) were also calculated. It revealed that lower OCN, lower FINS, and higher FPG were independently associated with carotid atherosclerosis (standardized *β*=-0.024, OR=0.976, 95%, CI: 0.941-1.012, p=0.013, for OCN; standardized *β*=-0.065, OR=0.937, 95%, CI: 0.891 -0.984, p=0.010, for FINS; standardized *β*=0.538, OR=1.713, 95%, CI: 1.163-2.522, p=0.006, for FPG).

### 3.3. Comparisons between the AS Patients and Non-AS Patients among Hypertension Subgroups and T2DM Subgroups

Since hypertension and T2DM were reported to be closely associated with the atherosclerosis, comparisons between the patients with AS or not among such two diseases subgroups were conducted.

Among the hypertension patients (n=116), the DBP of the AS patients (n= 51) was significantly higher than that of non-AS patients (n=65) (83.45±10.83 versus 82.31±8.73, p=0.035), but the difference was very small. Interestingly, the FINS of the AS patients was significantly lower than that of non-AS patients (11.20±5.74 versus 13.70±7.66, p=0.038). On the other hand, among the nonhypertension patients (n=219), the FPG of the AS patients (n= 85) was significantly higher than that of non-AS patients (n=134) (5.70±1.48 versus 5.19±0.84, p<0.001). The FBG of the AS patients was also significantly higher than that of non-AS patients (7.07±3.10 versus 6.59±1.97, p=0.016). The HbA1c of the AS patients was also significantly higher than that of non-AS patients (5.83±0.86 versus 5.56±0.55, p=0.002).

Among the patients with T2DM (n=50), the OCN of the AS patients (n= 25) was significantly lower than that of non-AS patients (n=25) (14.13±18.37 versus 18.36±8.70, p=0.028). Interestingly, the total cholesterol of the AS patients was significantly lower than that of non-AS patients (4.64±1.21 versus 4.73±0.84, p=0.047). On the contrary, among the patients without T2DM (n=285), the FPG of the AS patients (n= 111) was significantly higher than that of non-AS patients (n=134) (5.33±0.84 versus 5.11±0.70, p=0.030). The FINS of the AS patients was significantly lower than that of non-AS patients (10.05±5.28 versus 10.98±6.64, p=0.030).

## 4. Discussion

In the present study, we assessed OCN levels in 335 patients including 136 cases with carotid atherosclerosis cases and 199 control cases. Our study revealed that decreased OCN had a close relationship with the carotid atherosclerosis among middle-aged male patients.

In recent years, an increasing number of studies revealed that bones did not only act as a structural support, but also as an important endocrine organ. As an osteoblast-secreted small peptide, OCN was able to bound to hydroxyapatite in bone constitution and was also reported to be the active form of glucose and fat metabolism. For example, a lower level of OCN was found to decrease the glucose tolerance [17], which was consistent with our findings that OCN had negative significant Spearman correlation with HbA1c (r = -0.306, p <0.001). Similarly, this was reflected by the finding that, among the T2DM patients, the OCN of AS patients was significantly lower than that of non-AS patients. The mechanism by which OCN could regulate the glucose metabolism remained to be totally clarified. On one hand, it may be related to the fact that OCN could retard vascular endothelial cells apoptosis through this insulin-sensitizing effect [18], by stimulating the phosphatidylinositol 3-kinase (PI3-kinase)/Akt signaling pathway [19]. On the other hand, OCN could improve insulin resistance by decreasing inflammation and promote insulin signaling as well as the expression of Slc2a4/GLUT4 [20].

Not only was OCN found to be closely related to the glucose metabolism, but such influence was always accompanied with atherosclerotic changes. Abnormal expression of OCN was found to be related to both carotid atherosclerosis and diabetes metabolism. In our study, although there was no significant difference between the number of persons with type 2 DM in AS group and control group, the FPG and HbAlc were significantly different in two groups (p= 0.004 and p=0.008, respectively). Moreover, Pennisi et al. [21] previously found that the decline of OCN was closely related to the carotid lesion in patients compared with healthy group, with half of the subjects having diabetes. In an another Chinese study, OCN was independently associated with carotid atherosclerosis in patients who had impaired glucose regulation [22]. Such phenomenon might be explained by the fact that the early stage of atherosclerosis development is characterized by the endothelial apoptosis and subsequent endothelial dysfunction [23], and endothelial function could be impaired by high plasma concentration of free fatty acid (FFA) [24]. The vascular endothelium sharing the same pathway involves the insulin metastasis [18]. Therefore, through the aforementioned PI3-kinase dependent manner, OCN could promote the phosphorylation of endothelial cells' secretions and prevent its insulin-stimulated changes, thus decreasing the FFA-induced apoptosis of vascular endothelial cells [18].

The incurrence of the carotid atherosclerosis is associated with elevation of blood pressure, and each 20-mmHg in SBP or 10-mmHg DBP was associated with more than twofold increased risk of cardiovascular death, including carotid atherosclerotic events [25]. Since hypertension is strongly associated with endothelial function, whose dysfunction could lead to abnormal expression of vasoactive substances and induce dysfunctional contraction or dilation of blood vessels, it is suspected that there could be a potential relationship between OCN and SBP/DBP. In our study, we found no significant correlation between SBP/DBP and OCN, and there is no significant difference between the number of hypertension patients in AS group and control group. Such finding was similar to the previous human study conducted by Xu et. al. [26], but was inconsistent with their mice study which indicated that exogenous OCN significantly reduced the mean blood pressure level and promoted vascular endothelium-dependent relaxation of the aortic arch [27]. It is not clarified how OCN influences the blood pressure in human and thus more investigations are needed.

In our study, CRP and HDL-C were found to be significantly different in AS group and control group, and CRP was also found to be independently and inversely related to OCN. This was similar to the findings by Lombardo et. at. [28] that elevated CRP might indicate carotid plaque instability, and predict the presence of atherosclerosis [3]. However, despite the fact that OCN might be the potential link between carotid atherosclerosis and inflammation (CRP), the mechanism by which OCN reflects the changes of CRP in the atherosclerotic pathology remains to be unknown, and future investigations are needed to make it clarified.

Our study has some limitations. First, we did not differentiate OCN into carboxylated OCN (cOCN) and uncarboxylated OCN (unOCN) and make comparisons between them. cOCN was reported to be decarboxylated in the acidic environment during active bone resorption and released into circulation [29], while unOCN could not be secreted into circulation due to its reduced binding affinity for bone minerals in vitamin K environment [30]. However, some clinical studies indicated that both cOCN and unOCN were associated with energy metabolism and atherosclerosis [31]. For example, Ogawa-Furuya et. al reported that the ucOCN levels were inversely related to the abdominal aortic calcification score in men [32]. On the other hand, others reported that vitamin K could accelerate the transference of uncarboxylated osteocalcin to carboxylated osteocalcin [33], and thus the ratio of such two types of osteocalcin could be influenced and reflected by the current diet vitamin K intake [34]. Second, we did not include the individual thickness of CIMT in each patient or stratify its thickness to investigate the potential relationship between its thickness and OCN level and other parameters. Luo and his colleagues found that the age, SBP, FPG, FINS, and CRP were independently associated with C-IMT in a metabolically healthy Chinese population [35]. Finally, since our study is a cross-sectional study, it is not clear whether the abnormal expression of OCN is the causal factor of carotid atherosclerosis. Therefore, more prospective studies are necessary to address such issue.

In summary, we confirmed that the serum OCN level was inversely associated with the carotid atherosclerosis in middle-aged male adults. Due to the significant correlation between OCN and FPG, PBG, and HbAlc, the effects of OCN on atherosclerosis might be closely related to its ability to improve glucose and lipid metabolism.

## Figures and Tables

**Table 1:** Demographic and clinical characteristics of patients.

Markers	Means ± SD			p value*∗*
	AS group	Control group	Total	
Number	136	199	335	
Age, years	52.45 ± 3.09	52.32 ± 4.80	52.4 ± 3.87	0.546
OCN	18.71 ± 6.20	20.38 ± 7.19	19.71 ± 6.85	0.039
SBP, mmHg	125.22 ± 15.23	123.24 ±13.98	124.04 ± 14.53	0.332
DBP, mmHg	80.39 ± 10.25	80.34 ± 9.59	80.36 ± 9.85	0.755
BMI, kg/m^2^	25.41 ± 2.84	25.77 ± 2.85	25.62 ± 2.85	0.438
FPG, mm/dl	5.81 ± 1.52	5.32 ± 0.98	5.52 ± 1.25	0.004
PBG, mm/dl	7.47 ± 3.40	6.85 ± 2.41	7.10 ± 2.86	0.259
HbAlc, %	5.88 ± 0.88	5.63 ± 0.67	5.73 ± 0.77	0.008
FINS	10.05 ± 5.41	11.56 ± 6.89	10.94 ± 6.36	0.076
Calcium, mg/dl	2.41 ± 0.14	2.40 ± 0.14	2.41 ± 0.14	0.586
Phosphorus, mg/dl	1.13 ± 0.16	1.12 ± 0.14	1.13 ± 0.15	0.647
Total cholesterol, mg/dl	4.69 ± 0.98	4.86 ± 0.91	4.79 ± 0.94	0.068
Triglyceride, mg/dl	2.00 ± 1.45	2.04 ± 1.35	2.02 ± 1.39	0.440
HDL-C, mg/dl	1.07 ± 0.25	1.15 ± 0.31	1.11 ± 0.29	0.030
LDL-C, mg/dl	2.60 ± 0.80	2.67 ±0.77	2.64 ± 0.78	0.336
BUN, mg/dl	5.35 ± 1.09	5.22 ± 1.16	5.27 ± 1.13	0.198
Creatinine, mg/dl	72.06 ± 10.63	74.85 ± 14.26	5.27 ± 1.33	0.085
CRP, mg/l	4.08 ± 1.55	3.93 ± 1.71	3.99 ± 3.82	0.041

SBP, systolic blood pressure; DBP, diastolic blood pressure; BMI, body mass index; FPG, fasting plasma glucose; PBG, postprandial blood glucose; HDL-cholesterol, high-density lipoprotein cholesterol; LDL-cholesterol, low-density lipoprotein cholesterol; BUN, blood urea nitrogen; CRP, C-reaction protein.

**Table 2:** The comparisons of hypertension, T2DM, and smokers in AS and control group.

Items	AS group 136	Control group 199	Total	P value
Hypertension	51 (37.5%)	65 (32.7%)	116 (34.6%)	0.361
Type 2 DM	25 (18.4%)	25 (12.6%)	50 (14.9%)	0.190
Smokers	57 (41.9%)	69 (34.7%)	126 (37.6%)	0.179

DM: diabetes mellitus

**Table 3:** Correlations of OCN with various clinical and biochemical markers in whole study population.

Markers	r	p
SBP	-0.056	0.303
DBP	-0.088	0.109
BMI	-0.085	0.122
Waistline	-0.090	0.102
FPG	-0.311	<0.001
PBG	-0.272	<0.001
FINS	-0.091	0.096
Cr.	0.125	0.022
TG	-0.061	0.267
TC	0.026	0.630
HDL-C	0.074	0.176
LDL-C	0.140	0.010
CRP	-0.107	0.049
HbA1c	-0.266	<0.001

## Data Availability

The data used to support the findings of this study are available from the corresponding author upon request.
